# Hypertrophic Cardiomyopathy and Atrial Fibrillation: A Review

**DOI:** 10.7759/cureus.21101

**Published:** 2022-01-11

**Authors:** Vishnu Palyam, Ahmad T Azam, Oladipo Odeyinka, Rasha Alhashimi, Sankeerth Thoota, Tejaswini Ashok, Ibrahim Sange

**Affiliations:** 1 Internal Medicine, Jagadguru Jayadeva Murugarajendra Medical College, Davanagere, IND; 2 Internal Medicine, Allama Iqbal Medical College, Lahore, PAK; 3 Internal Medicine, University of Ibadan College of Medicine, Ibadan, NGA; 4 Internal Medicine, University of Baghdad College of Medicine, Baghdad, IRQ; 5 Internal Medicine, Meenakshi Medical College Hospital and Research Institute, Kancheepuram, IND; 6 Internal Medicine, Jagadguru Sri Shivarathreeshwara Medical College, Mysore, IND; 7 Internal Medicine, K.J. Somaiya Medical College, Mumbai, IND

**Keywords:** atrial fibrillation, obstructive cardiomyopathy, left atrial ablation, atrial fibrillation management, hypertrophic cardiomyopathy

## Abstract

Hypertrophic cardiomyopathy (HCM) is an inherited cardiological condition that exhibits various clinical symptoms. The leading cause of atrial fibrillation (AF) in patients with HCM is advanced diastolic dysfunction and left atrial dilatation and remodeling. In addition to the gradual symptomatic and functional decline caused by AF, there is an increased risk of thromboembolic disease and mortality, especially if there is a rapid ventricular rate or obstruction of the left ventricular outflow tract. The mainstay of management of AF in HCM is a combination of non-pharmacological lifestyle and risk factor modification, long-term anticoagulation, and rhythm control with anti-arrhythmic medications, septal ablation, and radiofrequency catheter ablation. This article has examined the development of AF in HCM, its clinical symptomatology, and its impact, highlighting its management and the mortality associated with AF in HCM.

## Introduction and background

Hypertrophic cardiomyopathy (HCM) is an umbrella term for a variegated heart disease defined by left ventricular hypertrophy (LVH) in the absence of any aberrant cardiac etiologies like hypertension or aortic stenosis [1]. Vulpian took the first step in developing the diagnosis of HCM by identifying it anatomically in a patient in the year 1868 [2]. According to statistics, 1 out of 500 people is affected by HCM, with a large percentage of patients being undiagnosed, making it one of the major causes of cardiac mortality in the US [3]. The prevalence of HCM exhibits a higher inclination toward the African population and is predominantly observed in males of younger ages [4]. HCM is considered to be a complex disorder that tends to manifest in patients with attributable risk factors such as a pre-existing arrhythmia, presence of strong family history, the occurrence of an abnormal blood pressure response on exercise, and ubiquity of deteriorating heart function [5]. Being a predicament of inheritance, up to 60% of cases of HCM are caused due to mutations in sarcomeric proteins such as myosin binding protein C, beta-myosin light chain, troponin I, and troponin T. In comparison, 5-10% of them are caused due to other genetically linked neuromuscular disorders like Friedreich's ataxia and mitochondrial myopathies [6]. The symptoms capitalized to make a provisional diagnosis of the disease may vary with individuals and mainly involve chest pain, fatigue, syncope, and exertion dyspnea causing little exercise [7]. To reach an adequate diagnosis, the most appropriate investigations used for HCM include electrocardiogram (ECG) and echocardiography (ECHO), which are performed and interpreted together due to their ability to provide complementary information [1]. A ≥13 mm increase in ventricular septal thickness in the absence of secondary determinants like hypertension and aortic stenosis is habitually considered to be diagnostic of HCM [1]. Other investigating modalities also include ambulatory monitoring, exercise ECG, and magnetic resonance imaging (MRI) [1]. The comprehensive management of HCM starts with a systematic approach of three-generation family history, which incorporates a regular physical examination, a routine ECG, and a conventional ECHO of first-degree relatives [8]. Other therapeutic interventions encompass lifestyle modifications such as discontinuation of high-energy sports and use of first-line medications such as beta-blockers and calcium-channel blockers [9]. Implantable cardiac defibrillator and septal reduction therapy should be considered in patients with significant risk factors and severe left ventricular outflow tract (LVOT) obstruction, respectively [9]. The management goal in HCM is to screen families of patients with positive family history, alleviate symptoms of dyspnea and chest pain, improve the cardiac function to prevent hypertrophic adversities, advance the scrutinizing modalities to achieve an early diagnosis in young adults, hinder the chances of premature sudden cardiac deaths, impede the development of future complications, repress events of hospitalizations, and improve the prognostication of HCM itself [9]. Being heterogeneous, HCM embraces a spectrum of manifestations depending on the extent and severity of the disease [10]. Atrial fibrillation (AF) is the most common arrhythmia that tends to sustain in patients with HCM, with the majority of them developing poor tolerance to it [10]. AF reduces LV filling time, predisposing the patient to an advanced diastolic dysfunction, left atrial dilatation, and extensive cardiac remodeling [10]. AF occurring in patients with HCM commences thromboembolic events, especially in the setting of ventricular tachycardia or LVOT obstruction, reduces the cardiac output, increases hemodynamic instability, and exacerbates a progressive symptomatic and functional decline with a heightened rate of cardiac mortality and morbidity [11]. This review article explores the clinical interrelation between HCM and AF and highlights the diagnostic and therapeutic possibilities.

## Review

Pathogenic mechanisms and hemodynamic implications

**Figure 1:**
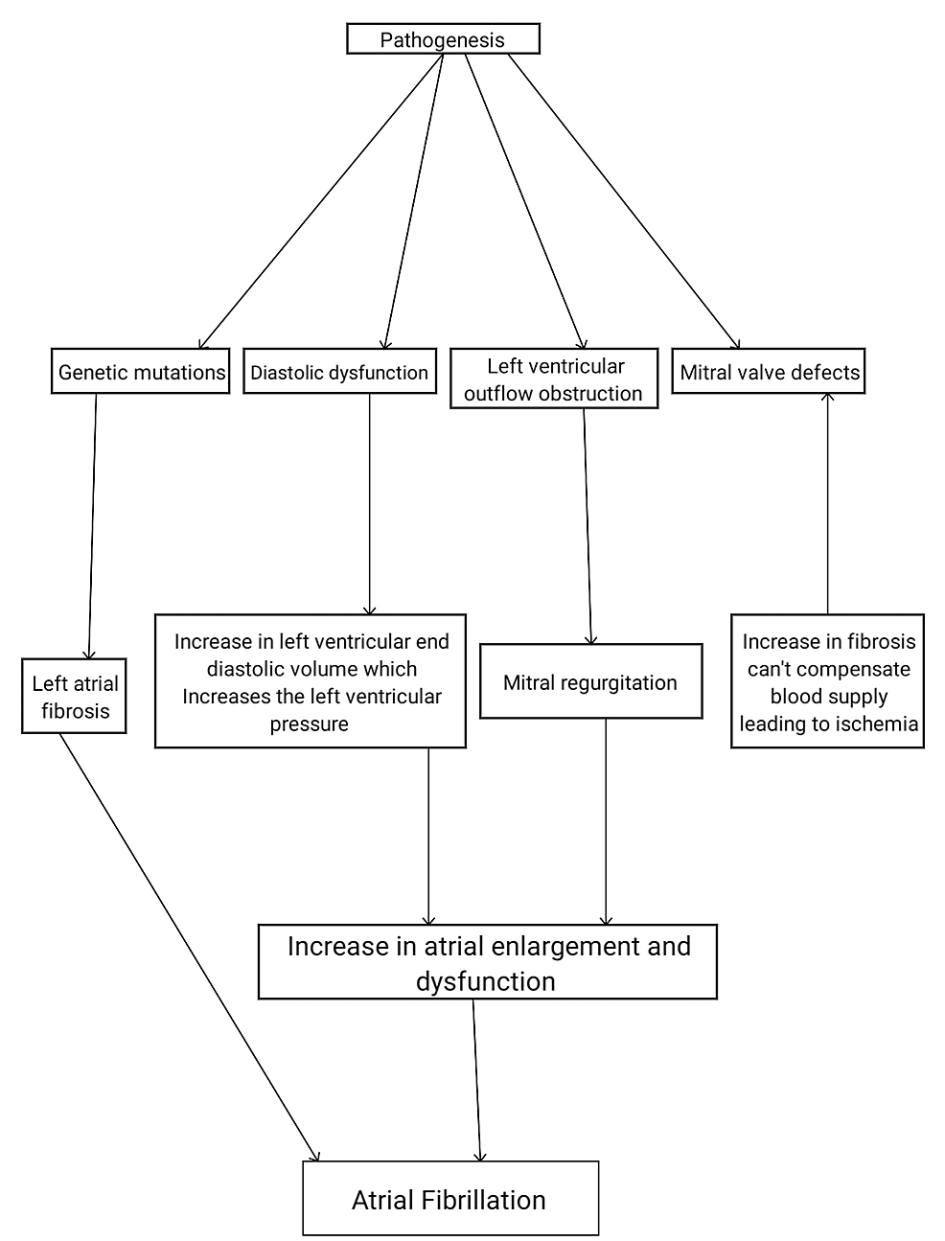
Pathogenesis of atrial fibrillation in hypertrophic cardiomyopathy

The pathogenesis of HCM is explained by a mutation that causes structural changes in sarcomere proteins, which results in an increased muscle cell size of the heart [1]. The mutations element a disarrangement of cells, which precipitate fibrosis in the structural components of the cardiac muscle cells, augmenting LVH and an increased thickness of the interventricular septum [12]. Histopathologically, the cardiomyocytes show complete disorganization and fibrosis, characterized by the loss of nuclei and accumulation of extracellular connective tissue [13]. These changes precipitate as LVOT obstruction, which leads to an elevated LV end-systolic volume in the systolic phase (contraction stage) (Figure 1). A failure to meet the circulation needs augments tissue ischemia and the leaflet of the valve, causing mitral regurgitation (MR) [14]. This disease can be characterized as a single-gene disorder with an autosomal-dominant inheritance pattern, and one allele mutation is usually enough to cause the disease, even though its expression and penetrance can vary considerably [15]. Statistically, the disease is shown to be inherited by about 60% of patients with HCM [15]. On the other hand, diastolic dysfunction (the relaxation phase) ensues due to an increased thickness in the LV wall inducing the need for an intensified blood supply [16]. Other inheritance modes, apart from autosomal dominant and X-linked, have been shown to exist but are rare in occurrence [16]. Both phenocopy and syndromic conditions have been shown to contribute to the development of HCM [16,17]. The syndromic conditions mainly comprise Noonan syndrome and storage disorders like Anderson-Fabry's disease.

In contrast, the phenocopy condition includes Fabry's disease, which is associated with an X-linked pattern of inheritance [16-18]. Pioneering studies conducted by Geisterfer-Lowrance et al. led to the discovery of an essential variant of MYH7 gene that coded for a sarcomere protein called myosin heavy chain (MYH7), thereby attempting to uncover the molecular genetic basis of HCM, which came to become the basis of large-scale discoveries made in the following years [1,19]. There were a small number of autosomal-dominant mutations that have led to premature truncation of the encoded proteins, which primarily include the p.Gln425X mutation in the MYBPC3 gene or the c.2864-2865delCT in the MYBPC3 gene and explained as a gain of a stop codon or frameshift mutation in itself [20,21]. The nonsense-mediated decay (NMD) pathway identifies the premature termination codon (PTC), which precipitates the release of the elongation factors (proteins that attach amino acids to the template during protein synthesis), and the decay-inducing complex (involved in mRNA degradation) [22,23]. Consequently, degradation of such transcripts ensues and causes a decline in the number of functional proteins [24]. The absence of a natural stop codon in a transcript induces a mechanism called the "non-stop decay pathway," which identifies the transcripts and releases them from ribosomes to prevent translation [24,25]. Furthermore, the exosome complex degrades the released transcripts while the No-Go mRNA decay pathway stalls ribosome progression during translation, resulting in an endo-nucleolytic cleavage of the mRNA transcript [23]. As a result of these quality control mechanisms, truncated proteins fail to synthesize, causing the transcripts containing PTC, which escape the NMD, to be expressed as truncated proteins resulting in abnormal proliferation of the myocardial septum [1].

A hypertrophied LV typically has an average end-diastolic volume, a standard or elevated ejection fraction (65-70%), and a decreased end-systolic volume [25]. When hyperdynamic ejection occurs, the anterior leaflet of the mitral valve moves anteriorly during systole, passing against the hypertrophied interventricular septum during that time, which results in obstruction of outflow in approximately one-third of patients with HCM [26]. HCM is characterized by dynamic blocks, which are further determined by factors such as the ventricular preload, afterload, and myocardial contractility itself [26-30]. According to a research study analyzing LVOT obstruction conducted by Maron MS et al., out of 320 patients who were diagnosed with HCM and suffered from a predominantly obstructive disease that was characterized by LV outflow gradients, 225 of them presented with symptoms of heart failure, which was detectable by exercise alone [30]. Diastolic dysfunction is considered the most common cause of heart failure among HCM patients [31]. The interstitial fibrosis and increased stiffness lead to thickening of the ventricular wall, which augments slowed relaxation, LA volumes elevation, and an eventual prediction of pathological developments such as AF and heart failure themselves [31]. The disease contributes to developing mechanisms that hinder the heart's normal automaticity, leading to an increased prevalence of AF in HCM patients [32]. HCM elements LV thickness, impedes chamber contractility, and promotes conduction abnormalities in the left atrium [32]. Automaticity is primarily determined by atrial muscle cells' action potential maintained by the physiological amalgamation of an inward rectifier K^+^ current and pacemaker current (I^f^) [33]. Since the resting potential is stabilized by a high flow of K^+^ ions balanced by a high pacemaker current, a decrease in the inward flow of K^+^ ions and already elevated pacemaker current hampers the automaticity leading to an aberrant conduction system [33]. Early afterdepolarizations are caused mainly by prolongation of action potential duration because of allowing the L-type Ca^2+^ current to recover from inactivation, causing depolarizing and inward movement of Ca^2+^ ions. These changes in the atrial muscle cell contribute to the prevalence of AF in patients diagnosed with congenital long-QT syndrome [34]. Ryanodine receptors (RyRs) are specialized sarcoplasmic/endoplasmic reticular channels that are known to precipitate the abnormal release of calcium, resulting in delayed afterdepolarizations [35]. A combination of transmembrane abnormalities and calcium overload in the sarcoplasmic reticulum causes premature opening of RyRs. It induces the sodium-calcium (Na^+^-Ca^+^) pump activation, which exchanges three Na^+^ ions for one Ca^2+^ leading to a net depolarizing inward positive-ion movement [35]. These changes lead to delayed afterdepolarizations and a discrepancy in the automaticity leading to the development of AF [35].

The extracellular matrix primarily comprises small, spindle-shaped cells known as fibroblasts [36]. Up to 75% of cardiac cells are made up of these cells, but they account for less than 10% of total cardiac mass [36]. Profibrotic stimuli activate an aberrant differentiation of fibroblasts into myofibroblasts [37]. Myofibroblasts tend to be secretory and nonproliferative, contribute to the formation of contractile scar, and are shown to possess smooth muscle actin, which is associated with increased differentiation into these cells [37]. Bone marrow-derived cells, endothelial cells, pericytes, and endothelial cells contribute to the sources that explain the origination of fibroblasts and myofibroblasts in the remodeled myocardium [37].

Several pathways have been identified to enhance the molecular and cellular mechanisms contributing to atrial fibrosis development. The calcium entry pathway contributes to extracellular signal-regulated kinase (ERK) phosphorylation and activation, consequently inducing fibroblast survival and promoting fibrosis [38,39]. A cardiomyocyte-fibroblast coupling enables low-resistance electrical junctions, which modulate mutual electrical activity and account for slowed conduction and the implementation of reentrant arrhythmias [40,41]. There is evidence that physiological effects of fibrosis on atrial conduction occur as a consequence of collagen production that physically separates cardiomyocytes instead of fibroblasts that couple with cardiomyocytes and electrically load them [42]. Maintenance of AF in HCM is mainly dependent on the up-regulation of inward rectifier currents, which Girmatsion Z et al. explained, who conducted a study on 62 patients undergoing mitral valve repair or bypass grafting [42]. Thirty-one of 62 patients were diagnosed with AF, and the study helped provide potential therapeutic benefits and new insights attempting to explain molecular mechanisms for AF maintenance in HCM patients [42,43].

**Figure 2:**
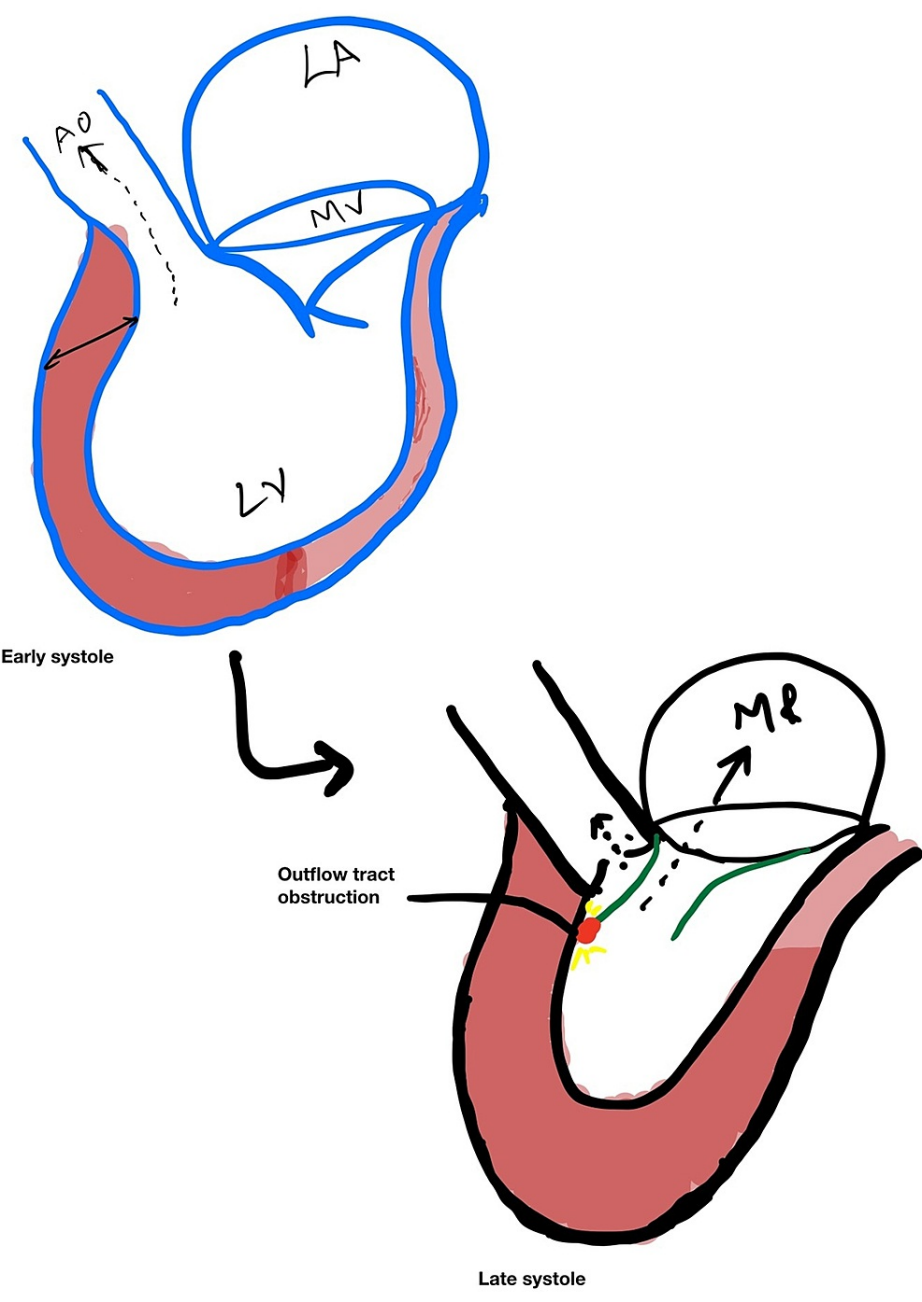
Mitral regurgitation with an increase of thickening of the left ventricular wall LA, left atrium; MR, mitral regurgitation; Ao, aorta; MV, mitral valve; LV, left ventricle

An increase in the thickness of the LV wall and interventricular septum obstructs the aorta and causes an increase in LV diastolic volume and pressure because the movement of the mitral valve anteriorly causes MR (Figure 2).

Risk factors and the clinical impact for the development of AF in HCM

According to various research studies, diagnostic modalities such as the ECG and ECHO have been successful in describing independent factors associated with AF in patients with HCM [44]. LA diameter, age, and heart failure class have shown to be the strongest independent predictors of the development of AF [45]. One of the most critical risk factors for the development of AF in HCM patients is LA size [44]. A cohort study performed by Olivotto I et al. in 480 patients of HCM (age at diagnosis >50 years) in the year 2001 that involved a nine-year follow-up concluded that there was a significant association between HCM and the LA size with 107/480 patients developing AF as a consequential complication of the disease [44]. A meta-analysis conducted by Guttmann OP et al. in 2013 in 7381 patients of HCM attempted to understand the prevalence of AF in HCM by explaining the association of LA size with the disease, only to reveal LA diameters of 45 mm and 38 mm in patients with AF and sinus rhythm, respectively [45]. The development of paroxysmal atrial fibrillation (PAF) was shown to have an imperative association with LA volume and left atrial volume index (LAVI) [46]. While LA volume was shown to be the most sensitive and specific marker for detecting PAF, LAVI was established to provide additional information on LA modeling, proving a stronger predictor of AF development than right atrial modeling in patients diagnosed with HCM [46]. In a study conducted by Tani T et al. in 141 patients of HCM, an LAVI >40 mL/m^2^ was considered a crucial prognostic tool in determining the development of AF in these patients [46].

Age is considered one of the most indicative predictors of AF in HCM patients [44]. According to several cohort studies, AF associated with HCM is rarely seen to develop in patients aged <30 years and is more prominent in the older groups [44]. Age thresholds ranging from 40 to 50 years have proven to be vital indicators of AF development in the general population and patients with HCM themselves [44]. LA enlargement is shown to occur due to the amalgamation of obstructive physiology, myocardial stiffness, irregular heart rhythms, and MR, with MR being one of the most critical repercussions associated with AF in patients with HCM [47]. Some studies have also shown that a New York Heart Association Class III/IV, moderate to severe MR, and an LV ejection fraction less than 50% are associated with an elevated risk of AF [47]. Siontis KC et al. conducted a retrospective analysis of >3500 patients with HCM from 1975 to 2012, and studies concluded that several echocardiographic markers in HCM patients like LV wall thickness and LV stiffness are associated with higher chances of developing AF as a consequence of structural abnormalities precipitated by the disease [47]. Inhomogeneous atrial conduction is determined by P wave dispersion (PWD), which is used as an estimation modality to assess PAF development in patients with HCM [48]. Early measurement of the LA diameter and PWD could lead to an early identification of high-risk patients of HCM prone to develop AF, allowing the establishment of appropriate treatments and follow-up protocols required for long-term management of the disease [48]. Ozdemir O et al. (2004) conducted a study in 30 patients from 1998 to 2001. With a sensitivity of 96% and specificity of 91% for detecting AF, as measured by PWD greater than 52.5 ms of the P wave, results showed 27 patients with AF compared with a control group consisting of 53 patients without any documented AF episodes and a significant correlation between PWD and AF in HCM patients [48].

AF can be defined as either paroxysmal when it can be terminated spontaneously or successfully cardioverted to sinus rhythm, or as chronic when it can be established, based on ECG recordings obtained, following the acute onset of symptoms or by chance during a routine medical examination [44]. A study performed by Olivotto I et al. in 2001 in 480 patients found that ischemic stroke was eight times as typical in the AF group as it was in the control group (21% AF; 2.6% non-AF), with most of the patients being affected by the symptoms of progressive heart failure [44]. In a study conducted by Rowin EJ et al. in the Tufts Medical Center Hypertrophic Cardiomyopathy Institute from 2004 to 2014, 1558 patients showed that 226/304 patients experienced symptomatic AF, which highlighted an impairment in the quality of life (QOL) due to the unpredictability of the frequency and timing of the transient episodes of AF [49]. HCM patients with AF tend to have an increased risk of death by up to four-fold compared to individuals with sinus rhythm, with a significant amount of mortality associated with spontaneous episodes of thromboembolism (TE) and worsening heart failure [44]. Moreover, few sudden cardiac deaths have also been reported due to deterioration of AF into ventricular tachycardia, especially when pre-excitation is present [44]. In HCM, AF is common and is associated with a high risk of TE [45]. Most data suggest that anticoagulation should be considered for AF patients as there is critical evidence proving that AF increases the risk of systemic TE in HCM patients [45]. Guttmann OP et al. conducted a retrospective longitudinal cohort study from 1986 and 2008. They concluded that there is an eight-fold risk of TE in HCM patients with AF, and the development of TE is considered a poor predictive factor in the long-term prognostication of HCM [45,50]. AF in patients with HCM continues to sway the etiologic diagnosis among physicians and should be investigated thoroughly.

Management

Several studies have identified that lifestyle modifications like healthy eating and physical activity could reduce the incidence of AF and produce successful ablation outcomes. Even though these have not explicitly included HCM patients, recommendations and treatment of underlying comorbidities like diabetes, hypertension, and sleep apnea should be undertaken to prevent AF.

Pharmacological Treatment

1. AV node suppressants

Beta-adrenergic antagonists have long been used in the treatment of HCM. Beta-blockers were believed to reduce symptoms (primarily breathlessness and chest pain) and enhance exercise tolerance mainly by decreasing the heart rate and a consequent lengthening of diastole. A clinical study conducted by Cohen LS et al. in 13 patients with beta-adrenergic antagonist (propranolol) results showed that 10 patients had improvement with the symptoms of obstruction blockade and hemodynamics in ischemic hearts, and also when propranolol was used in exercise-induced angina pectoris in people with ischemic heart disease, there was a significant improvement in symptoms [51]. Another small study was performed by Thompson DS et al. with propranolol (beta-adrenergic antagonist) in 13 HCM patients aged 18-62 years. Results have shown that there is a decrease in oxygen consumption in these patients. Indirectly the reduction in heat rate is beneficial in severe ischemic patients to prevent excessive oxygen consumption [52]. Calcium-channel blockers serve as a cornerstone in AV node suppression therapy and have been used for a long time. A study was performed using a calcium-channel blocker (verapamil) by Hanrath P et al. in 11 HCM patients. The results revealed that there was a significant improvement in regional LV relaxation and LV filling, 10 minutes after intravenous injection (IV) of verapamil (0.15 mg/kg body weight), and prolonged isovolumic LV relaxation time was observed. The data concluded that intravenously administered verapamil could significantly shorten the LV relaxation time in HCM patients, and it is associated with improved filling of the left ventricle [53]. Another study was performed by TenCate FJ et al. using verapamil in 10 HCM patients using a combined hemodynamic-ultrasonic technique. LV pressure was recorded with the help of ECHO. These patients were given IV verapamil for a short term, and data show no significant improvement after short-term administration. LV relaxation and filling dynamics were abnormal [54].

Harrison DC et al. conducted a clinical study in 15 patients between 8 and 42 years with a new beta-adrenergic blocker called Nethalide. These patients underwent right heart catheterization after beta-adrenergic receptor blocking with Nethalide, and five postoperative subjects also underwent the procedure. The heart rate and rate of pressure development in the right ventricle decreased slightly during rest, but the cardiac index was not altered by Nethalide administration. The study concluded that there was a significant symptomatic relief in these HCM patients [55].

A study performed by Pollick C et al. in 43 HCM patients showed substantial decreases in basal subaortic pressure gradient in 35 patients and a subgroup of 13 patients despite prolongation of the pre-ejection period from 104 to 137 ms, and the cardiac output remained unchanged after the use of disopyramide. So despite a decrease in contractility, cardiac output appears to be maintained because disopyramide reduces mitral valve anterior motion resulting in decreased MR. The study concluded that in patients with HCM and resting obstruction of LV outflow, disopyramide produced predictably favorable hemodynamic effects [56].

Digoxin was considered adequate for controlling AF rate; however, it does not effectively manage ventricular rate when exercised. Digoxin is a positive inotropic drug beneficial in patients with heart failure. A study performed by Roberts SA et al. in 115 HCM patients with AF has given digoxin in Technology Advancement Center of the University Hospital Consortium (UHC), with a rapid ventricular rate (>120 beats/minute). The time to control the rate is 11.6 h to bring the heart rate to <100 beats/minute. The study concluded that rapid ventricular rate is inconsistent, inefficient, and in some cases inappropriate [57]. However, the use of digoxin is limited by the unpredictability of its adverse reactions like arrhythmias and gastrointestinal symptoms.

2. Control of AF in HCM patients

The management of AF was based on two principles -- symptom relief (managed with rate control or rhythm control methods) and prevention of complications (prevented with anticoagulation). Acute complications include strokes, tachycardia-induced cardiomyopathy, and worsening heart failure.

Control of AF in HCM patients leads to marginally lower rates of cardiovascular deaths, decreased number of admissions to hospitals for heart failure, lesser thromboembolic events, severe bleeding, pacemaker implantation, or adverse effects from antiarrhythmic drugs in the general population. In a large randomized trial performed by Al-Khatib SM in 28,836 HCM patients, the study concluded that after treatment with antiarrhythmic drugs, there was a significant decrease in mortality and a symptomatic improvement in these HCM patients [58]. There are about four classes of antiarrhythmic drugs (Table 1), Class 1 (sodium-channel blockers) and Class 3 (potassium-channel blockers) are helpful for rhythm control, and Class 2 (beta-blockers) and Class 4 (calcium-channel blockers) are for rate control. These drugs are used depending upon the progression of the disease and the clinical outcome.

**Table 1:** Classification of antiarrhythmic drugs

Classification	Mechanism
Class I	Sodium-channel blockers (Intermediate associated/dissociation)
Class II	Beta-blockers
Class III	Potassium-channel blockers (sotalol is also a beta-blocker; amiodarone has Class I, II, III, IV activity)
Class IV	Calcium-channel blockers

Class III antiarrhythmic medications were often used due to significant hypertrophy of the left ventricle and proarrhythmic potential. Amiodarone is an effective agent, even though it was commonly used despite having a relatively young population. In a study performed by Malasana G et al. in 52 consecutive patients with HCM, 24 developed AF, and 29 (63%) were restored to sinus rhythm with amiodarone after a five-year follow-up period, which resulted in sinus rhythm being maintained in 22 out of 29 patients. Results concluded that following treatment with amiodarone, these patients required fewer alterations in their drug therapy, experienced a small number of embolic episodes, and needed fewer cardioversions [59]. In another small study performed by McKenna WJ et al. with ambulatory ECG monitoring (48- hour), amiodarone was found to be effective in treating paroxysmal AF/supraventricular tachycardia in eight of nine patients without provoking ventricular arrhythmias, but discontinued due to side effects in three patients, one with hair loss, one with facial discoloration, and one with neurological symptoms. After discontinuing amiodarone in three patients due to side effects, the drug was reintroduced at a lower dose and was well tolerated. They concluded that with the use of amiodarone, there was a significant decrease in paroxysmal AF/supraventricular tachycardia in HCM patients [60].

Generally, sotalol is the antiarrhythmic choice for otherwise young patients with AF in HCM. HCM patients may also benefit from sotalol in preventing AF. In a study performed by Tendera M et al. in 30 patients with HCM, six out of seven patients experienced improvement by the abolition of supraventricular arrhythmias by sotalol. In comparison, seven of 13 patients experienced improvement through suppression of arrhythmias. After six months of follow-up, 25 patients had better exercise tolerance. Ventricular tachycardia was abolished in four of eight cases. However, it appeared during sotalol treatment in one case free of repetitive arrhythmias on placebo. It should be administered under supervision to monitor QT prolongation over the first few doses. Monitoring of renal function, ECG changes, serum potassium, and serum magnesium was regularly done when compared with placebo. But in one patient, it had to be discontinued due to bronchospasm. The study concluded that sotalol significantly improves exercise tolerance and effectively suppresses both supraventricular and ventricular arrhythmias in patients with HCM [61].

Dofetilide is a Class III antiarrhythmic agent. In a study performed by Moore JC in 1404 HCM patients, 32% female patients were reviewed retrospectively from 2008 to 2012 at the Cleveland Clinic to suppress the AF. Twenty-one patients were discharged on dofetilide, three discontinued during loading due to QTc prolongation, and one was ineffective. The study concluded that among this group of patients with AF and HCM, dofetilide was well tolerated, and it helped to manage AF in 21/25 (84%) cases [62].

3. Thromboembolic prophylaxis

Patients with HCM who have AF have a substantial TE risk. Major guidelines recommend advising patients to take anticoagulants for a long term to prevent TE in HCM patients with AF.

Thrombotic events are insufficiently appreciated complications of HCM, which causes a stroke or other peripheral vascular events or may lead to sudden cardiac death. In a clinical study conducted by Maron BJ et al. in 900 patients who were followed up for seven years, 51 (6%) patients experienced a stroke or other vascular events. Out of these, 21 patients (41%) died. The results concluded that non-anticoagulated patients with AF had a significantly higher cumulative incidence of these events than the warfarin-treated patients (31% vs 18%) [63] (Table 2). In a retrospective analysis performed by Robinson K et al. between 1957 and 1985 in 174 HCM patients with AF who were followed up for 11 years, 25 of the 52 patients were treated with conventional therapy alone and seven with amiodarone alone, and the remaining 20 patients received standard treatment first. The study concluded that there was a prominent decrease in the episodic reoccurrence of TE and a maintenance of sinus rhythm, and the use of amiodarone therapy was associated with the maintenance of sinus rhythm, fewer drug therapy adjustments, and fewer attempts at direct current cardioversions (over a shorter period) [64] (Table 2).

**Table 2:** Summary of studies of different designs conducted between 1990 and 2019 on thromboembolic prophylaxis of AF in HCM AF, atrial fibrillation; HCM, hypertrophic cardiomyopathy; NOACs, non-vitamin K antagonist oral anticoagulants; n, number

Reference	Therapy	No of patients	Study design	Result
Maron BJ et al. (2002) [63]	Warfarin	900	43.2% of patients with AF were anticoagulated	Cumulative incidence of thromboembolism among non-anticoagulated patients was twice that of patients anticoagulated (31% vs 18%)
Robinson K et al. (1990) [64]	Amiodarone	174	45 of 52 patients with AF received conventional therapy alone, 25 patients were followed with amiodarone, and seven patients with only amiodarone	Fewer alterations in drug therapy, fewer embolic episodes, and more remained in sinus rhythm; fewer current cardioversion attempts have been noted
Higashikawa M et al. (1997) [65]	Warfarin	83	37% (seven out of 19) of HCM patients with AF were treated with warfarin	Ischemic stroke was seen in six out of seven warfarin-treated HCM patients with AF
Kitaoka H et al. (2001) [66]	Warfarin	91	45% of patients with AF anticoagulated	42% of patients without warfarin vs 10% with warfarin experienced thromboembolism
Jung H et al.(2019) [67]	Effectiveness and safety of non-vitamin K antagonist vs warfarin (oral anticoagulants)	2459	A cohort study conducted in The Korean National Health Insurance Service database warfarin-treated group of patients with HCM and AF (n = 955) who were compared with a 1:2 propensity-matched NOACs-treated group (n = 1,504)	All-cause mortality and composite fatal cardiovascular events were lower than those taking warfarin and suggest that patients with HCM and AF can be safely and effectively treated with NOACs

The ischemic cerebrovascular events are more commonly seen in HCM patients with AF than in patients with only HCM. The risk of sudden cardiac death incidence increases with this. Anticoagulation with warfarin reduces the thrombus formation and decreases the occurrence of cerebrovascular thrombotic events. Higashikawa M et al. performed a clinical study at Shiga University of Medical Science Hospital from 1976 to 1994. In 83 patients with HCM, 19 of them were diagnosed with AF, and seven patients from these 19 were treated with warfarin. The results showed that six out of these seven warfarin-treated HCM patients with AF had decreased thromboembolic risk. The study concluded a significant improvement with warfarin for preventing cerebrovascular thrombotic events [65] (Table 2). In a small study conducted by Kitaoka H et al. in 91 Japanese HCM patients, the patients were treated with warfarin and the prognostic value of AF for long-term mortality and morbidity was examined. Eighty-seven patients with AF had 12 cardiovascular events (embolic events in five, heart failure in four, and sudden death in three), and four patients without AF had one cardiovascular event (embolic event in one). The study results concluded that 42% of patients without warfarin vs 10% with warfarin experienced TE, and there was a significant improvement in decreasing thrombotic events in HCM patients with AF [66] (Table 2).

Alternatives to warfarin include non-vitamin K antagonist oral anticoagulants (NOACs). A cohort study was performed by Jung H et al. in 2458 HCM patients from the Korean National Health Insurance Service database with a warfarin-treated group of patients with HCM and AF (n = 955) who were compared with a 1:2 propensity-matched NOAC-treated group (n = 1504). The study concluded that the risk of stroke and major bleeding was similar in patients with HCM and AF taking NOACs. However, the all-cause mortality and composite fatal cardiovascular events were lower than those taking warfarin, suggesting that HCM and AF patients can be safely and effectively treated with NOACs [67] (Table 2).

Nonpharmacological Treatment

Patients with recurrence of AF, drug resistance, and contraindications to pharmacological therapy are surgical candidates. 

Di Donna P et al. performed a study in 61 HCM patients with paroxysmal or long-standing AF. After radiofrequency catheter ablation (RFCA), 41 patients were restored to sinus rhythm. An approach with pulmonary vein (PV) isolation plus linear lesions was employed in these patients. Most patients with long-term sinus rhythms who underwent RFCA had symptomatic improvement. However, redo procedures were necessary in some cases. The study results concluded a significant decrease in AF with RFCA in HCM patients [68].

Another study was performed clinically by Bunch TJ et al. in 33 patients with HCM, in which the patients underwent PV isolation or wide-area circumferential ablation and were followed up for two years with 12-lead ECG, 24-hour ambulating ECGs, ECHO, event monitor strips, and SF 36 QOL surveys being obtained before ablation and for routine follow-up. Additional linear ablation might improve outcomes in patients with severe LA enlargements and more advanced diastolic dysfunction. The study concluded that there were a prominent reduction in AF reoccurrence and reasonable symptomatic control in HCM patients who had undergone ablation and PV isolation [69].

A clinical study was performed by Walters T et al., in which 83 AF patients with age <75 years were followed up for 12 months; these patients underwent an early catheter ablative intervention, and after the procedure was done, they were followed up with detailed baseline assessment including blood pressure readings, anthropometric measurements, digital ECGs, and transthoracic ECHO, allowing evaluation of myocardial strain by two-dimensional speckle tracking ECHO. And after effective AF ablation, with a negligible AF burden over 12 months, the study concluded that there was a significant improvement in reverse structural remodeling in HCM patients and there was a decrease in the occurrence of AF [70]. Derek P et al. performed a study in 30 patients who were followed up for 12 months. Cavotricuspid isthmus block and PV isolation were performed on all patients.

Additionally, patients suffering from persistent and long-standing AF, and those who had undergone repeated procedures, were created with linear lesions in the left atrium, and complex, fragmented atrial potentials were ablated. The study concluded that it was a safe and an effective therapeutic option to treat paroxysmal AF patients with HCM. Ablation worked less effectively in patients with persistent AF and even less effectively in long-term continuous AF. Patients often required repeated procedures. Antiarrhythmic drug therapy is often needed because of a significant degree of atrial remodeling [71]. AF can be treated by circumferentially isolating all PVs or isolating electrical connections within their Ostia. Walczak F et al. conducted a study in 80 HCM patients (51 men, 29 women) with AF, who were symptomatic and drug-resistant. Selective ablation was done in those patients who had detectable AF. The results were obtained with success in 61 patients, and there was a significant clinical improvement in another nine patients. The study concluded that selective ablation effectively eliminated AF with only a few triggering foci. However, chronic and persistent AF patients experienced some delay in benefiting from the treatment due to the delayed reversal of atrial remodeling [72].

Dilling-Boer D et al. performed a study in 47 HCM patients (32 men and 15 women) with a mean age of 47 ± 10 years and who were followed up for seven months. Initially, only ectopic foci were ablated, and a relatively high recurrence rate was associated with trigger-directed ablation of focally induced AF. Study results concluded that comprehensive ablation techniques separate all PVs empirically and decrease the LA structural remodeling in HCM patients and there was a significant decrease in AF occurrence in these patients [73]. However, more extensive ablation techniques have emerged that isolate all PVs empirically and place circumferential ablation lines in the left atrium. 

In AF recurrence, the posterior wall of the left atrium plays a key role, but the advantages of LA posterior wall isolation are unclear. HE X et al. performed a meta-analysis in 594 AF patients and compared posterior wall ablation with other ablation groups. The study participants had a lower atrial arrhythmia recurrence rate (relative risk 0.81, 95% confidence interval 0.68-0.97, p = 0.02). The study concluded that there was a significant decrease in the reoccurrence of AF without any procedure-related complications [74]. 

Limitations

HCM has a complex network of etiologies with multiple underlying components. This article concentrates solely on HCM's impact on AF for analysis and does not address other variables in the prognosis of this condition.

## Conclusions

As evident from the studies reviewed in this article, HCM causes structural changes in myocardial tissue, leading to the formation of AF. The occurrence of AF during the evolution of HCM is common, but at the same time, it is associated with a poor prognosis. The causes of its development are multifactorial, including the typical anatomical and hemodynamic alterations associated with HCM and genetic factors. Also, AF arises from mechanical and electrical atrial adverse remodeling processes, particularly progressive LA dilation. Clinical practitioners should be on the lookout for this arrhythmia during regular follow-ups with patients. Several echocardiographic markers in HCM patients, like LV wall thickness and LV stiffness, are associated with higher chances of developing AF due to structural abnormality precipitated by the disease. The clinical implication of difficulties that physicians confront when dealing with these illnesses was the reason for the development of various strategies for the treatment. Nowadays, several pharmacological and nonpharmacological strategies are available for treating acute AF, preventing recurrences, and controlling the rate of patients with permanent AF. Beta-blockers and amiodarone, and interventional catheter-based radiofrequency ablation in some cases, are options to maintain the patients in sinus rhythm. Disopyramide was indicated as a third-line agent to be used for the treatment of life-threatening arrhythmia and AF. Other management strategies such as regular monitoring via ambulatory electrocardiogram for those without diagnosed AF and radiofrequency catheter ablation for HCM patients with AF were previously reported to be associated with more minor embolic events. Moreover, anticoagulation is a cornerstone in treating HCM patients already after the first documented AF episode. Anticoagulation with warfarin reduces the thrombus formation and decreases the occurrence of cerebrovascular thrombotic events. Alternatives to warfarin including NOACs are the mainstay for mortality, and composite fatal cardiovascular events were lower than in those taking warfarin. Such treatment protocols must be individually adjusted for each patient. Finally, we believe that more significant research into the link between HCM and AF is needed to develop a more coordinated and direct approach to diagnosing and treating these disorders.

## References

[1] Marian AJ, Braunwald E (2017). Hypertrophic cardiomyopathy: genetics, pathogenesis, clinical manifestations, diagnosis, and therapy. Circ Res.

[2] Vulpian A (1868). Contribution à l’étude des rétrécissements de l’orifice ventriculo-aortique. Arch Physiol.

[3] Husser D, Ueberham L, Jacob J (2018). Prevalence of clinically apparent hypertrophic cardiomyopathy in Germany-an analysis of over 5 million patients. PLoS One.

[4] Eberly LA, Day SM, Ashley EA (2020). Association of race with disease expression and clinical outcomes among patients with hypertrophic cardiomyopathy. JAMA Cardiol.

[5] Pellnitz C, Geier C, Perrot A, Dietz R, Osterziel KJ, Haverkamp W (2005). [Sudden cardiac death in familial hypertrophic cardiomyopathy. Identification of high-risk patients]. Dtsch Med Wochenschr.

[6] Ho CY (2012). Genetic considerations in hypertrophic cardiomyopathy. Prog Cardiovasc Dis.

[7] Geske JB, Ommen SR, Gersh BJ (2018). Hypertrophic cardiomyopathy: clinical update. JACC Heart Fail.

[8] Gersh BJ, Maron BJ, Bonow RO (2011). 2011 ACCF/AHA Guideline for the Diagnosis and Treatment of Hypertrophic Cardiomyopathy: a report of the American College of Cardiology Foundation/American Heart Association Task Force on Practice Guidelines. Developed in collaboration with the American Association for Thoracic Surgery, American Society of Echocardiography, American Society of Nuclear Cardiology, Heart Failure Society of America, Heart Rhythm Society, Society for Cardiovascular Angiography and Interventions, and Society of Thoracic Surgeons. J Am Coll Cardiol.

[9] Teekakirikul P, Zhu W, Huang HC, Fung E (2019). Hypertrophic cardiomyopathy: an overview of genetics and management. Biomolecules.

[10] Vaidya K, Semsarian C, Chan KH (2017). Atrial fibrillation in hypertrophic cardiomyopathy. Heart Lung Circ.

[11] Bosch NA, Cimini J, Walkey AJ (2018). Atrial fibrillation in the ICU. Chest.

[12] Brigden W (1987). Hypertrophic cardiomyopathy. Br Heart J.

[13] Varnava AM, Elliott PM, Mahon N, Davies MJ, McKenna WJ (2001). Relation between myocyte disarray and outcome in hypertrophic cardiomyopathy. Am J Cardiol.

[14] Firth J (2019). Cardiology: hypertrophic cardiomyopathy. Clin Med (Lond).

[15] Greaves SC, Roche AH, Neutze JM, Whitlock RM, Veale AM (1987). Inheritance of hypertrophic cardiomyopathy: a cross sectional and M mode echocardiographic study of 50 families. Br Heart J.

[16] Branzi A, Romeo G, Specchia S (1985). Genetic heterogeneity of hypertrophic cardiomyopathy. Int J Cardiol.

[17] Kozor R, Grieve SM, Tchan MC (2016). Cardiac involvement in genotype-positive Fabry disease patients assessed by cardiovascular MR. Heart.

[18] Elliott P, Baker R, Pasquale F, Quarta G, Ebrahim H, Mehta AB, Hughes DA (2011). Prevalence of Anderson-Fabry disease in patients with hypertrophic cardiomyopathy: the European Anderson-Fabry Disease survey. Heart.

[19] Geisterfer-Lowrance AA, Kass S, Tanigawa G, Vosberg HP, McKenna W, Seidman CE, Seidman JG (1990). A molecular basis for familial hypertrophic cardiomyopathy: a β cardiac myosin heavy chain gene missense mutation. Cell.

[20] Niimura H, Patton KK, McKenna WJ, Soults J, Maron BJ, Seidman JG, Seidman CE (2002). Sarcomere protein gene mutations in hypertrophic cardiomyopathy of the elderly. Circulation.

[21] van Dijk SJ, Dooijes D, dos Remedios C (2009). Cardiac myosin-binding protein C mutations and hypertrophic cardiomyopathy: haploinsufficiency, deranged phosphorylation, and cardiomyocyte dysfunction. Circulation.

[22] Popp MW, Maquat LE (2016). Leveraging rules of nonsense-mediated mRNA decay for genome engineering and personalized medicine. Cell.

[23] Siwaszek A, Ukleja M, Dziembowski A (2014). Proteins involved in the degradation of cytoplasmic mRNA in the major eukaryotic model systems. RNA Biol.

[24] Vasudevan S, Peltz SW, Wilusz CJ (2002). Non-stop decay--a new mRNA surveillance pathway. Bioessays.

[25] Frischmeyer PA, van Hoof A, O'Donnell K, Guerrerio AL, Parker R, Dietz HC (2002). An mRNA surveillance mechanism that eliminates transcripts lacking termination codons. Science.

[26] Braunwald E, Lambrew CT, Rockoff SD, Ross J Jr, Morrow AG (1964). Idiopathic hypertrophic subaortic stenosis. I. A description of the disease based upon an analysis of 64 patients. Circulation.

[27] Maron MS, Olivotto I, Zenovich AG (2006). Hypertrophic cardiomyopathy is predominantly a disease of left ventricular outflow tract obstruction. Circulation.

[28] Shah JS, Esteban MT, Thaman R (2008). Prevalence of exercise-induced left ventricular outflow tract obstruction in symptomatic patients with non-obstructive hypertrophic cardiomyopathy. Heart.

[29] Braunwald E, Oldham HN Jr, Ross J Jr, Linhart JW, Mason DT, Fort L 3rd (1964). The circulatory response of patients with idiopathic hypertrophic subaortic stenosis to nitroglycerin and to the Valsalva maneuver. Circulation.

[30] Braunwald E, Ebert PA (1962). Hemodynamic alterations in idiopathic hypertrophic subaortic stenosis induced by sympathomimetic drugs. Am J Cardiol.

[31] Guttmann OP, Pavlou M, O'Mahony C (2015). Prediction of thrombo-embolic risk in patients with hypertrophic cardiomyopathy (HCM Risk-CVA). Eur J Heart Fail.

[32] Paur H, Wright PT, Sikkel MB (2012). High levels of circulating epinephrine trigger apical cardiodepression in a β2-adrenergic receptor/Gi-dependent manner: a new model of Takotsubo cardiomyopathy. Circulation.

[33] Stillitano F, Lonardo G, Zicha S, Varro A, Cerbai E, Mugelli A, Nattel S (2008). Molecular basis of funny current (If) in normal and failing human heart. J Mol Cell Cardiol.

[34] Johnson JN, Tester DJ, Perry J, Salisbury BA, Reed CR, Ackerman MJ (2008). Prevalence of early-onset atrial fibrillation in congenital long QT syndrome. Heart Rhythm.

[35] Yeh YH, Wakili R, Qi XY (2008). Calcium-handling abnormalities underlying atrial arrhythmogenesis and contractile dysfunction in dogs with congestive heart failure. Circ Arrhythm Electrophysiol.

[36] Yue L, Xie J, Nattel S (2011). Molecular determinants of cardiac fibroblast electrical function and therapeutic implications for atrial fibrillation. Cardiovasc Res.

[37] Travers JG, Kamal FA, Robbins J, Yutzey KE, Blaxall BC (2016). Cardiac fibrosis: the fibroblast awakens. Circ Res.

[38] Olson ER, Shamhart PE, Naugle JE, Meszaros JG (2008). Angiotensin II-induced extracellular signal-regulated kinase 1/2 activation is mediated by protein kinase Cdelta and intracellular calcium in adult rat cardiac fibroblasts. Hypertension.

[39] Thum T, Gross C, Fiedler J (2008). MicroRNA-21 contributes to myocardial disease by stimulating MAP kinase signalling in fibroblasts. Nature.

[40] Miragoli M, Gaudesius G, Rohr S (2006). Electrotonic modulation of cardiac impulse conduction by myofibroblasts. Circ Res.

[41] Zlochiver S, Muñoz V, Vikstrom KL, Taffet SM, Berenfeld O, Jalife J (2008). Electrotonic myofibroblast-to-myocyte coupling increases propensity to reentrant arrhythmias in two-dimensional cardiac monolayers. Biophys J.

[42] McDowell KS, Vadakkumpadan F, Blake R, Blauer J, Plank G, Macleod RS, Trayanova NA (2013). Mechanistic inquiry into the role of tissue remodeling in fibrotic lesions in human atrial fibrillation. Biophys J.

[43] Girmatsion Z, Biliczki P, Bonauer A (2009). Changes in microRNA-1 expression and IK1 up-regulation in human atrial fibrillation. Heart Rhythm.

[44] Olivotto I, Cecchi F, Casey SA, Dolara A, Traverse JH, Maron BJ (2001). Impact of atrial fibrillation on the clinical course of hypertrophic cardiomyopathy. Circulation.

[45] Guttmann OP, Rahman MS, O'Mahony C, Anastasakis A, Elliott PM (2014). Atrial fibrillation and thromboembolism in patients with hypertrophic cardiomyopathy: systematic review. Heart.

[46] Tani T, Tanabe K, Ono M (2004). Left atrial volume and the risk of paroxysmal atrial fibrillation in patients with hypertrophic cardiomyopathy. J Am Soc Echocardiogr.

[47] Siontis KC, Geske JB, Ong K, Nishimura RA, Ommen SR, Gersh BJ (2014). Atrial fibrillation in hypertrophic cardiomyopathy: prevalence, clinical correlations, and mortality in a large high-risk population. J Am Heart Assoc.

[48] Ozdemir O, Soylu M, Demir AD (2004). P-wave durations as a predictor for atrial fibrillation development in patients with hypertrophic cardiomyopathy. Int J Cardiol.

[49] Rowin EJ, Hausvater A, Link MS (2017). Clinical profile and consequences of atrial fibrillation in hypertrophic cardiomyopathy. Circulation.

[50] Guttmann OP, Pavlou M, O'Mahony C (2017). Predictors of atrial fibrillation in hypertrophic cardiomyopathy. Heart.

[51] Cohen LS, Braunwald E (1967). Amelioration of angina pectoris in idiopathic hypertrophic subaortic stenosis with beta-adrenergic blockade. Circulation.

[52] Thompson DS, Naqvi N, Juul SM, Swanton RH, Coltart DJ, Jenkins BS, Webb-Peploe MM (1980). Effects of propranolol on myocardial oxygen consumption, substrate extraction, and haemodynamics in hypertrophic obstructive cardiomyopathy. Br Heart J.

[53] Hanrath P, Mathey DG, Kremer P, Sonntag F, Bleifeld W (1980). Effect of verapamil on left ventricular isovolumic relaxation time and regional left ventricular filling in hypertrophic cardiomyopathy. Am J Cardiol.

[54] TenCate FJ, Serruys PW, Mey S, Roelandt J (1983). Effects of short-term administration of verapamil on left ventricular relaxation and filling dynamics measured by a combined hemodynamic-ultrasonic technique in patients with hypertrophic cardiomyopathy. Circulation.

[55] Harrison DC, Braunwald E, Glick G, Mason DT, Chidsey CA, Ross Jr JO (1964). Effects of the beta-adrenergic blockade on the circulation, with particular reference to observations in patients with hypertrophic subaortic stenosis. Circulation.

[56] Pollick C, Kimball B, Henderson M, Wigle ED (1988). Disopyramide in hypertrophic cardiomyopathy. I. Hemodynamic assessment after intravenous administration. Am J Cardiol.

[57] Roberts SA, Diaz C, Nolan PE (1993). Effectiveness and costs of digoxin treatment for atrial fibrillation and flutter. Am J Cardiol.

[58] Al-Khatib SM, Allen LaPointe NM, Chatterjee R (2014). Rate- and rhythm-control therapies in patients with atrial fibrillation: a systematic review. Ann Intern Med.

[59] Malasana G, Day JD, Bunch TJ (2009). Atrial fibrillation in hypertrophic obstructive cardiomyopathy - antiarrhythmics, ablation and more!. J Atr Fibrillation.

[60] McKenna WJ, Harris L, Rowland E, Kleinebenne A, Krikler DM, Oakley CM, Goodwin JF (1984). Amiodarone for long-term management of patients with hypertrophic cardiomyopathy. Am J Cardiol.

[61] Tendera M, Wycisk A, Schneeweiss A, Poloński L, Wodniecki J (1993). Effect of sotalol on arrhythmias and exercise tolerance in patients with hypertrophic cardiomyopathy. Cardiology.

[62] Moore JC, Trager L, Anzia LE (2018). Dofetilide for suppression of atrial fibrillation in hypertrophic cardiomyopathy: a case series and literature review. Pacing Clin Electrophysiol.

[63] Maron BJ, Olivotto I, Bellone P (2002). Clinical profile of stroke in 900 patients with hypertrophic cardiomyopathy. J Am Coll Cardiol.

[64] Robinson K, Frenneaux MP, Stockins B, Karatasakis G, Poloniecki JD, McKenna WJ (1990). Atrial fibrillation in hypertrophic cardiomyopathy: a longitudinal study. J Am Coll Cardiol.

[65] Higashikawa M, Nakamura Y, Yoshida M, Kinoshita M (1997). Incidence of ischemic strokes in hypertrophic cardiomyopathy is markedly increased if complicated by atrial fibrillation. Jpn Circ J.

[66] Doi Y, Kitaoka H (2001). Hypertrophic cardiomyopathy in the elderly: significance of atrial fibrillation. J Cardiol.

[67] Jung H, Yang PS, Jang E (2019). Effectiveness and safety of non-vitamin K antagonist oral anticoagulants in patients with atrial fibrillation with hypertrophic cardiomyopathy: a nationwide cohort study. Chest.

[68] Di Donna P, Olivotto I, Delcrè SD (2010). Efficacy of catheter ablation for atrial fibrillation in hypertrophic cardiomyopathy: impact of age, atrial remodelling, and disease progression. Europace.

[69] Bunch TJ, Munger TM, Friedman PA (2008). Substrate and procedural predictors of outcomes after catheter ablation for atrial fibrillation in patients with hypertrophic cardiomyopathy. J Cardiovasc Electrophysiol.

[70] Walters TE, Nisbet A, Morris GM (2016). Progression of atrial remodeling in patients with high-burden atrial fibrillation: implications for early ablative intervention. Heart Rhythm.

[71] Derejko P, Polańska M, Chojnowska L (2013). Catheter ablation of atrial fibrillation in patients with hypertrophic cardiomyopathy: atrial fibrillation type determines the success rate. Kardiol Pol.

[72] Walczak F, Szumowski L, Urbanek P (2006). Selective ablation or isolation of all pulmonary veins in atrial fibrillation -- when and for whom?. Kardiol Pol.

[73] Dilling-Boer D, Van Der Merwe N, Adams J (2004). Ablation of focally induced atrial fibrillation: selective or extensive?. J Cardiovasc Electrophysiol.

[74] He X, Zhou Y, Chen Y, Wu L, Huang Y, He J (2016). Left atrial posterior wall isolation reduces the recurrence of atrial fibrillation: a meta-analysis. J Interv Card Electrophysiol.

